# Refreshing memories

**DOI:** 10.7554/eLife.02041

**Published:** 2014-01-29

**Authors:** John E Lisman

**Affiliations:** 1 **John E Lisman** is in the Biology Department and Volen Center for Complex Systems, Brandeis University, Waltham, United Stateslisman@brandeis.edu

**Keywords:** CaMKII, subunit exchange, spread of activation state, single-molecule, holoenzyme, memory, *E. coli*

## Abstract

The exchange of CaMKII enzymes between larger structures called holoenzymes may provide the molecular mechanism underlying the long-term stability of memories.

**Related research article** Stratton M, Lee I-H, Bhattacharyya M, Christensen SM, Chao LH, Schulman H, Groves JT, Kuriyan J. 2014. Activation-triggered subunit exchange between CaMKII holoenzymes facilitates the spread of kinase activity. *eLife* **3**:e01610. doi: 10.7554/eLife.01610

**Image** A holoenzyme containing 12 CaMKII enzymes

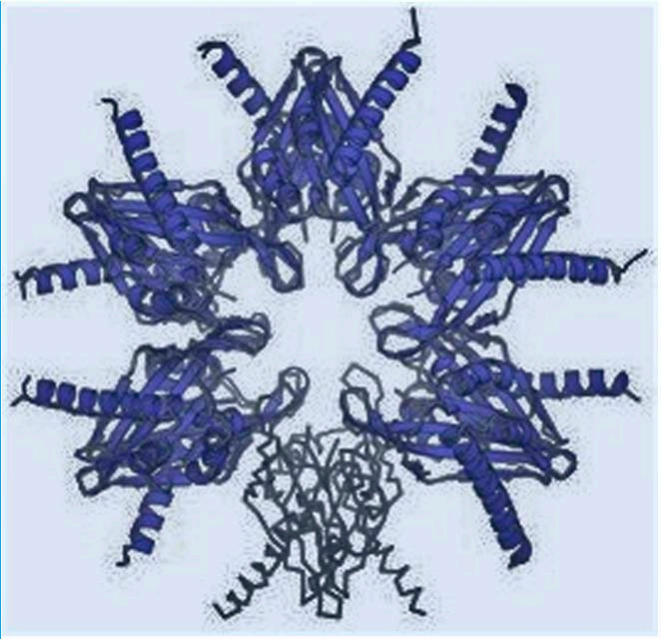

Although people complain about their memory when they cannot find their keys or recall the name of someone they have just met, the fact is that some memories can persist for a lifetime. What are the molecular mechanisms that our brains use to store such long-term memories, and how does this information remain so stable over time? Decades after an event, few of the molecules originally present will still be around, so the mechanism that stores a memory must allow newly synthesized molecules to be updated by molecules that already ‘contain’ the memory. Now, in *eLife*, Jay Groves, John Kuriyan and co-workers—including Margaret Stratton and Il-Hyung Lee of the University of California Berkeley as joint first authors—suggest a surprisingly simple view of how one of the leading candidates for memory storage—a complex called a CaMKII holoenzyme—might be updated (Stratton et al., 2014).

CaMKII holoenzymes contain 12 nearly identical molecules of an enzyme called CaM kinase II (CaMKII), arranged into two hexagonal rings, and they are found at the synapses that connect nerve cells with each other. CaMKII is activated when the arrival of a nerve impulse at the synapse causes an increase in the concentration of calcium ions within the cell. Previous work showed that CaMKII was strongly activated during a process called ‘long-term potentiation’ that is thought to underlie learning (Lee et al., 2009). This process—which is induced when two nerve cells are active at the same time—increases the strength of the synapse between the two nerve cells.

Once activated, each CaMKII subunit can phosphorylate neighbouring subunits in the holoenzyme in a process called autophosphorylation (Hanson et al., 1994). Of great interest is the fact that the autophosphorylation of a particular site—a threonine called T286—makes the enzyme persistently active, even after the calcium levels return to baseline (Miller and Kennedy, 1986). CaMKII can thus be considered as a ‘switch’ that remains ‘on’ until the enzyme becomes dephosphorylated by a phosphatase enzyme.

In the cytoplasm of the nerve cell, phosphatase activity is high and CaMKII becomes dephosphorylated within about a minute (Lee et al., 2009). However, during the induction of long-term potentiation, some activated CaMKII holoenzymes are relocated from the cytoplasm to a part of the synapse called the postsynaptic density. Within this structure, the rate of dephosphorylation of T286 is very low (Mullasseril et al., 2007). Moreover, should a T286 site become dephosphorylated, it is likely to be rapidly rephosphorylated by the autophosphorylation process described above. Thus, it is relatively easy to see how the ‘on’ state of CaMKII within the synapse could provide a persistent memory.

But that raises the issue of whether the turnover of these proteins might erase the memory encoded by such switches. If a CaMKII holoenzyme that had been activated was later destroyed when it became old, and was replaced with newly synthesized proteins, information might be lost. But what if turnover involved the replacement of a subunit rather than the removal of an entire holoenzyme? This scenario, first proposed by Marc Goldring and myself in an early computational model of CaMKII (Lisman and Goldring, 1988), has now been shown to be plausible by the work of Groves, Kuriyan and colleagues at UC Berkeley, the Lawrence Berkeley National Laboratory and Allosteros Therapeutics (Stratton et al., 2014).

Two groups of holoenzymes were labelled with fluorescent tags of different colours, and mixed to determine if subunits could be exchanged between different holoenzymes. Using optical techniques with high resolution, Stratton, Lee et al. could see single holoenzymes that contained both coloured tags—proof that subunits had indeed been swapped. So if this is the way that the production and degradation of these proteins are balanced in cells, the stability of information storage during protein turnover could occur as follows: when a phosphorylated subunit in an ‘on’ holoenzyme is replaced by an unphosphorylated subunit, this new subunit is autophosphorylated by the other subunits, restoring the holoenzyme to its full ‘on’ state (Figure 1).

**Figure 1.**
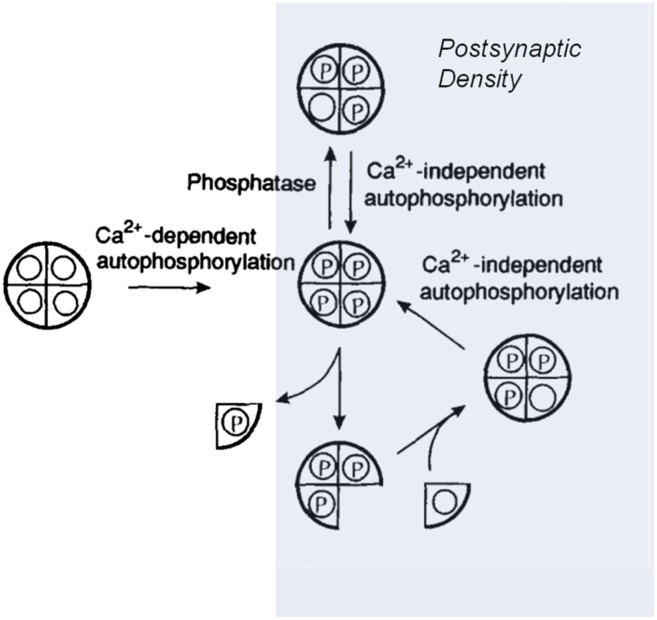
Model showing how the CaMKII switch remains ‘on’ despite protein turnover. A CaMKII holoenzyme (left, shown here with only 4 of its 12 subunits) in the cytoplasm of a cell is generally unphosphorylated (denoted by the open circles), because the level of phosphatase activity is high. During the induction of long-term potentiation, the concentration of calcium ions (Ca^2+^) rises, leading to activation and autophosphorylation of CaMKII (denoted by the letter P). Some of these molecules relocate to the postsynaptic density (shaded area on right) where the phosphatase activity is very low; this means that autophosphorylation (see main text) can keep CaMKII fully phosphorylated. In the subunit exchange observed by Stratton, Lee et al., a phosphorylated subunit can be replaced by an unphosphorylated one, which is then autophosphorylated by its neighbouring subunits, returning the holoenzyme to its fully phosphorylated ‘on’ state.

Figure credit: Adapted from Lisman J, Goldring M. 1988. Evaluation of a model of long-term memory based on the properties of the Ca^2+^/calmodulin-dependent protein kinase. *Journal de Physiologie* 83:187–197

We are all attracted to simplicity and beauty in science, and the CaMKII switch described above has these qualities. Perhaps subunit exchange, followed by autophosphorylation between subunits, is as central to memory storage as the base pairing of DNA is to genetic memory. However, there are several other viable candidates for molecular memory (Kandel, 2012; Tsien, 2013). Moreover, although CaMKII has been strongly implicated in maintaining long-term potentiation (Sanhueza et al., 2011), its role in maintaining memory has not been tested by looking at behaviour in animals. The gold standard for such tests is to make a memory and then determine whether this memory can be erased by an attack on the candidate memory molecule. In the mid 2000s, there was considerable excitement when another enzyme, called PKM-zeta, appeared to pass this test, but it was later shown that knocking out the gene for PKM-zeta had little effect on long-term potentiation or memory (Volk et al., 2013). It will be exciting to see in years to come which of the candidate memory molecules can pass this gold standard test.
